# Reward Behavior Disengagement, a Neuroeconomic Model-Based Objective Measure of Reward Pathology in Depression: Findings from the EMBARC Trial

**DOI:** 10.3390/bs13080619

**Published:** 2023-07-25

**Authors:** Michael A. Giles, Crystal M. Cooper, Manish K. Jha, Cherise R. Chin Fatt, Diego A. Pizzagalli, Taryn L. Mayes, Christian A. Webb, Tracy L. Greer, Amit Etkin, Joseph M. Trombello, Henry W. Chase, Mary L. Phillips, Melvin G. McInnis, Thomas Carmody, Phillip Adams, Ramin V. Parsey, Patrick J. McGrath, Myrna Weissman, Benji T. Kurian, Maurizio Fava, Madhukar H. Trivedi

**Affiliations:** 1 Department of Psychiatry, University of Texas Southwestern Medical Center, Dallas, TX 75390, USA; 2 Center for Depression Research and Clinical Care, Peter O’Donnell Jr. Brain Institute and Department of Psychiatry, University of Texas Southwestern Medical Center, Dallas, TX 75390, USAtracy.greer@utsouthwestern.edu (T.L.G.);; 3 Jane and John Justin Neurosciences Center, Cook Children’s Health Care System, Fort Worth, TX 76104, USA; 4 Department of Psychiatry, Harvard Medical School, Boston, MA 02215, USA; 5 McLean Hospital, Belmont, MA 02478, USA; 6 Department of Psychology, University of Texas at Arlington, Arlington, TX 76019, USA; 7 Department of Psychiatry and Behavioral Sciences, Stanford University, Stanford, CA 94305, USA; 8 Department of Psychiatry, University of Pittsburgh School of Medicine, Pittsburgh, PA 15213, USA; 9 Department of Psychiatry, University of Michigan School of Medicine, Ann Arbor, MI 48109, USA; 10 Peter O’Donnell Jr. School of Public Health, University of Texas Southwestern Medical Center, Dallas, TX 75390, USA; 11 Department of Psychiatry, Columbia University, New York, NY 10032, USA; 12 Department of Psychiatry and Behavioral Health, Stony Brook University Renaissance School of Medicine, Stony Brook, NY 11794, USA; 13 Massachusetts General Hospital, Boston, MA 02114, USA

**Keywords:** anhedonia, major depressive disorder, probabilistic reward task, treatment response, reward engagement

## Abstract

The probabilistic reward task (PRT) has identified reward learning impairments in those with major depressive disorder (MDD), as well as anhedonia-specific reward learning impairments. However, attempts to validate the anhedonia-specific impairments have produced inconsistent findings. Thus, we seek to determine whether the Reward Behavior Disengagement (RBD), our proposed economic augmentation of PRT, differs between MDD participants and controls, and whether there is a level at which RBD is high enough for depressed participants to be considered objectively disengaged. Data were gathered as part of the Establishing Moderators and Biosignatures of Antidepressant Response in Clinical Care (EMBARC) study, a double-blind, placebo-controlled clinical trial of antidepressant response. Participants included 195 individuals with moderate to severe MDD (Quick Inventory of Depressive Symptomatology (QIDS–SR) score ≥ 15), not in treatment for depression, and with complete PRT data. Healthy controls (*n* = 40) had no history of psychiatric illness, a QIDS–SR score < 8, and complete PRT data. Participants with MDD were treated with sertraline or placebo for 8 weeks (stage I of the EMBARC trial). RBD was applied to PRT data using discriminant analysis, and classified MDD participants as reward task engaged (*n* = 137) or reward task disengaged (*n* = 58), relative to controls. Reward task engaged/disengaged groups were compared on sociodemographic features, reward–behavior, and sertraline/placebo response (Hamilton Depression Rating Scale scores). Reward task disengaged MDD participants responded only to sertraline, whereas those who were reward task engaged responded to sertraline and placebo (*F*(1293) = 4.33, *p* = 0.038). Reward task engaged/disengaged groups did not differ otherwise. RBD was predictive of reward impairment in depressed patients and may have clinical utility in identifying patients who will benefit from antidepressants.

## 1. Introduction

Anhedonia, the loss of pleasure and/or reduced desire to pursue normally enjoyable activities, is a core symptom of major depressive disorder (MDD). Evidence for an anhedonic phenotype in patients with MDD is well supported. Notably, the probabilistic reward task (PRT), which measures implicit reward learning (i.e., the bias towards rewarding stimulus), has successfully measured reward learning impairments in MDD participants [1,2,3] as well as anhedonia-specific reward learning impairments [2,4,5]. However, attempts to validate the quantification of this anhedonic trait have not produced consistent findings [3,6,7,8]. Such shortcomings may, at least in part, be due to the omission of an empiric cost/benefit model from measures of reward impairment [9]. These economic models imply that the extent to which a person engages or disengages in the task at hand, and therefore, the magnitude of reward learning, is predicated on a favorable cost/benefit analysis of the reward being offered. Thus, reward task engagement is thought to be an important, yet overlooked component of the reward learning impairments seen in MDD.

### 1.1. Conceptualization of Reward Behavior Disengagement (RBD)

The current study aims to characterize the behavioral and clinical features of a novel economic index of reward task engagement called Reward Behavior Disengagement (RBD). RBD is built upon an economic conceptualization of hedonic behavior, namely, that the decision to engage in any activity is contingent on a favorable cost/benefit analysis, wherein the costs include, at minimum, the effort required to perform the activity well (see Supplementary Materials for RBD derivation and computational details). Clinically significant reward impairment, as examined from this perspective, can thus arise anytime the effort associated with a normally rewarding activity is overly penalized, and ultimately results in task disengagement. For example, in clinically healthy individuals, the dollar dropped onto the floor of one’s home is worth picking up, while the dollar left on the other side of the airport security checkpoint is not. However, in individuals with severe anhedonic impairment, as indexed by higher than normal RBD, neither dollar is worth the effort required to collect it.

### 1.2. Measuring RBD

It is worth noting that although this is the first work involving RBD, functionally equivalent measures can be independently derived by simply adapting the Integrated Signals and Economics (ISE) framework, formerly proposed by Lynn et al., to the reward task or behavior of interest (see Supplementary Materials for details on how this can be conducted). Thus, this work can be thought of as a real-world examination of the a priori ISE framework.

For this study, the ISE framework was applied to the PRT to produce our new measure, RBD. Briefly, the PRT involves two blocks. In block 1, participants experience asymmetric reward reinforcement among the two task stimuli; in block 2, participants process this experience to more effectively select rewarding stimuli. We hypothesized that RBD would differ between MDD and healthy control (HC) participants, specifically in block 2 after participants had been given the opportunity to process the asymmetric reinforcement schedule. Critically, and consistent with prior findings that blunted reward learning in the PRT is driven by abnormalities in anhedonic [2] MDD participants, we hypothesized that this difference would be driven by the presence of two distinct phenotypes within our MDD cohort: a reward task disengaged group (i.e., significantly elevated RBD relative to HCs), and an HC-like reward task engaged group. 

Finally, we tested the clinical utility of the reward task engaged and reward task disengaged classifiers by assessing their putative redundancy with sociodemographic and clinical features, as well as their prognostic significance on treatment outcomes. We hypothesized that the reward task engaged and reward task disengaged groups would differ in their response to an 8-week course of antidepressant treatment (sertraline) or placebo, as measured by the 17-item clinician-rated Hamilton Depression Rating Scale (HAMD-17) [10].

This report details our implementation of RBD in HC and MDD participants who were enrolled in the Establishing Moderators and Biosignatures of Antidepressant Response in Clinical Care (EMBARC) study [11]. To test our hypotheses, we addressed these specific questions:

1. Does RBD in block 2 differ between HC and MDD participants?
2. Among MDD participants, is there a level at which RBD is high enough to be considered objectively *disengaged* when compared to HC participants?
3. Do reward task engaged and reward task disengaged MDD participants differ in sociodemographic or clinical features?
4. Do reward task engaged and reward task disengaged MDD participants respond differently to sertraline versus placebo?

## 2. Materials and Methods

### 2.1. Design and Participants

The multisite two-phase, double-blind, EMBARC trial randomized 296 participants with MDD to sertraline or placebo (stage I) with the aim of identifying biosignatures of antidepressant response in MDD [11]. Additionally, the study included 40 HC participants who—as with the MDD group—were assessed at baseline and 1 week post baseline. This secondary analysis included 195 MDD and 40 HC participants with complete PRT data following the EMBARC protocol for model development using a randomly selected two-thirds of the total depressed cohort, with validation on the remaining one-third to occur at a later time. All participants were 18–65 years old. The study was approved by the Institutional Review Board at each site. All participants signed informed consent. See Table 1 for demographics.

MDD participants were diagnosed using the Structured Clinical Interview for DSM-IV (SCID) [12] and were not undergoing treatment for MDD. Participants were excluded if they scored <14 on the Quick Inventory of Depressive Symptomatology–Self Report (QIDS–SR) [13], if any other mental disorder was primary to MDD, if they had a lifetime history of an Axis-I mood disorder, psychotic disorder, or eating disorder, or did not score within the normal IQ range on the Wechsler Abbreviated Scale of Intelligence. For other exclusion criteria, refer to Trivedi et al. [11]. HC participants had no history of psychiatric diagnoses based on the SCID, and scored ≤8 on the QIDS–SR. Scores from the HAMD-17 [10] and the Snaith–Hamilton Anhedonia Scale [14] (SHAPS) were also collected to track MDD severity and measure anhedonia, respectively. 

### 2.2. Procedures

All data were collected at four sites: Columbia University (CU), Massachusetts General Hospital/McLean Hospital (MG), University of Michigan (UM), and University of Texas Southwestern Medical Center (TX). These data can be accessed on the National Institute of Mental Health Data Archive website (https://nda.nih.gov/edit_collection.html?id=2199, accessed on 30 April 2015).

#### 2.2.1. Probabilistic Reward Task (PRT)

PRT methods for EMBARC followed the methods of Pizzagalli et al. [3]. Briefly, participants viewed schematic faces with either a long or short mouth (Supplementary Figure S1). For each face presented, participants made one of two responses: (1) “long” when they judged the mouth to be the longer of the two stimuli, or (2) “short” when they judged the mouth to be the shorter one. The potential reward for correct identification was 20 cents. Correct identification of mouth length was not always rewarded, with one mouth length (rich stimulus) rewarded three times more frequently than the other (lean stimulus). Among HCs, this asymmetrical reinforcement schedule induces a response bias towards the rich stimulus (i.e., reward learning) [2,3,15,16,17]. Participants completed a practice phase, followed by 200 trials divided evenly into two blocks. 

#### 2.2.2. RBD Subgrouping of MDD Participants

Participants were categorized as either reward task engaged or reward task disengaged based on their block 2 RBD measurement (i.e., after they had been given the opportunity to process the asymmetric reinforcement schedule). In order to objectively specify an RBD score indicative of clinical impairment, quadratic discriminant analysis [18] was implemented to obtain a block 2 RBD cutoff value that most effectively distinguished the MDD group from the HC group (*p* < 0.05). This cutoff value was compared to the threshold of HC participants, which has been used previously to define the normal range of functioning in MDD participants [19]. 

### 2.3. Statistical Analyses

RBD scores between HC and MDD participants during block 2 were compared using a t-test with Cochran–Cox approximation to account for unequal variance. MDD participants were then classified into reward task engaged and reward task disengaged groups based on a threshold identified using HCs (described above). 

Baseline clinical and sociodemographic features were compared using chi-squared or t-tests. The features selected for comparison included age, sex, race, marital status, education level, employment status, number of depressive episodes (divided into three quantiles), length of current depressive episode (≤6 months, 7–24 months, >24 months), monthly income in USD (<2000, 2000–4000, >4000), number of medical comorbidities (divided into quartiles), as well as HAMD-17, QIDS–SR, and SHAPS scores. 

A 6 (time: 1, 2, 3, 4, 6, and 8 weeks) × 2 (treatment group: sertraline or placebo) × 2 (MDD RBD-subgroup: reward task engaged, reward task disengaged) repeated-measures linear mixed-model was conducted, and all interactions were used to test the moderator effect (differential prediction of treatment outcome with sertraline versus placebo) of RBD status as indicated by HAMD-17 scores across time. HAMD-17 scores were measured at baseline and weeks 1, 2, 3, 4, 6, and 8. Time was log-transformed, a random effect was assigned to each participant (with unstructured variance–covariance matrix), and a spatial power variance–covariance structure for the repeated measures error term was used. To control for baseline depression severity, baseline HAMD-17 scores were dropped from the model and entered as a covariate. A random intercept was used.

## 3. Results

### 3.1. Does RBD in Block 2 Differ between HC and MDD Participants?

*Yes.* In line with our hypothesis, block 2 RBD was significantly higher among MDD participants (*M* = 4.88, *SE* = 0.07, *n* = 195) than among HC participants (*M* = 4.64, *SE* = 0.05, *n* = 40) (*t*(91.9) = 2.00, *p* = 0.006).

### 3.2. Among MDD Participants, Is There a Level at Which RBD Is High Enough to Be Considered Objectively Impaired (or “Disengaged”) When Compared to HC Participants?

*Yes.* Quadratic discriminant analysis yielded a cutoff of 5.08 for block 2 RBD. Of the 195 participants with MDD, 137 (70.25%) had block 2 RBD < 5.08 (classified as reward task engaged), while 58 (29.75%) had block 2 RBD ≥ 5.08 (classified as reward task disengaged). A chi-square test revealed that the block 2 RBD cutoff criterion significantly differentiated the MDD and HC groups, ($\chi^{2}$(1, *n* = 235) = 5.03, *p* = 0.025). Of the 40 HCs examined, 35 were reward task engaged and only 5 were reward task disengaged. Sensitivity and specificity for meeting DSM-IV MDD criteria were 58.7%, (95% *CI* [53.5–64.1%]) and 88.9% (95% *CI* [76.0–96.3%]), respectively. See Supplementary Materials for the comparisons of RBD to traditional PRT measures. Please note however, that traditional PRT measures such as Response Bias and Discriminability share many closely-related computational variables with RBD, and these comparisons between these measures are difficult to interpret. 

### 3.3. Do Reward Task Engaged and Reward Task Disengaged MDD Participants Differ in Sociodemographic or Clinical Features?

*No*. Reward task engaged and reward task disengaged MDD participants did not differ on any sociodemographic or clinical feature (Table 2), which indicates that the RBD metric provides incremental information. Notably, reward task engaged and reward task disengaged MDD participants did not differ in self-reported anhedonia (SHAPS: *t*(103.5) = −0.73, *p* = 0.47) or depression severity (QIDS–SR: *t*(87.4) = −0.12, *p* = 0.91; HAMD-17: *t*(105.0) = −1.2, *p* = 0.27).

### 3.4. Do Reward Task Engaged and Reward Task Disengaged MDD Participants Respond Differently to Sertraline versus Placebo? 

**Figure 1:**
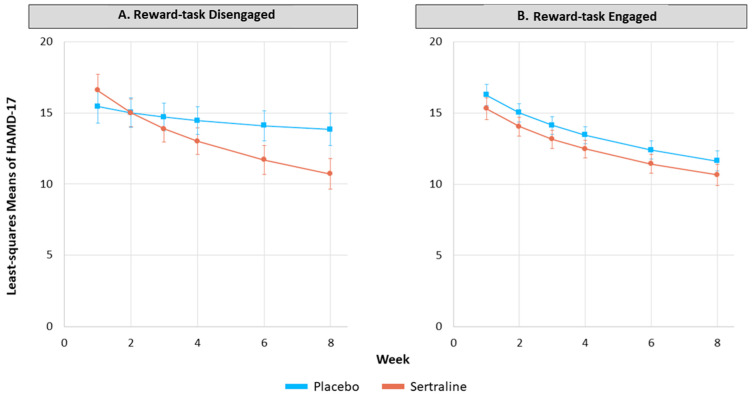
Effect of sertraline treatment on symptom severity among reward task disengaged (**A**) and reward task engaged (**B**) participants with major depressive disorder. Note: Shown with standard error bars; 17-item clinician-rated Hamilton Depression Rating Scale scores (HAMD-17); *F*(1293) = 4.33, *p* = 0.038.

*Yes*. There was a significant 3-way interaction, 7 (time) × 2 (treatment) × 2 (RBD group); (*F*(1293) = 4.33 *p* = 0.038). To visualize, we plotted the changes in HAMD-17 scores with sertraline and placebo separately for the reward task engaged and reward task disengaged groups. As shown in Figure 1B, HAMD-17 scores of the reward task engaged group did not differ between placebo and sertraline treatments over time. However, in the reward task disengaged group (Figure 1A), the rate of change in HAMD-17 scores differed significantly between the sertraline and placebo treatment groups, with marked separation observed by week 6. Specifically, those MDD patients who were reward task disengaged had greater reductions in their HAMD-17 scores as treatment progressed when on sertraline compared to placebo.

## 4. Discussion

In this study, we describe for the first time a newly developed novel measure of reward impairment, Reward Behavior Disengagement or RBD, and demonstrate its clinical significance. To our knowledge, this is the first study to utilize a neuroeconomic approach to identify an objective, clinically relevant measure of reward impairment, especially in the context of a placebo-controlled clinical trial of MDD. Unlike current symptom measures such as the SHAPS, RBD is an objective measure of reward impairment, thereby directly addressing the issue of the heterogeneity of anhedonic pathology seen in depression [6,8]. RBD differed significantly between HC and MDD participants, and discriminant analysis identified a subgroup of MDD participants for whom RBD was elevated beyond that seen in HCs. Finally, the clinical importance of RBD is highlighted by the moderating effect of the RBD depression classifiers reward task disengaged and reward task engaged on acute-phase treatment outcomes, an effect which was independent of socioeconomic or clinical features.

Reward impairment as described by the SHAPS and RBD differ fundamentally from each other, and SHAPS scores were not predictive of RBD classification. Anhedonia, characterized in an economic framework, can arise when the effort associated with a normally rewarding activity is overly penalized, and ultimately results in the decision to not engage in the activity. This over-penalization of costs is fairly easy to objectively quantify (e.g., RBD). In contrast, the SHAPS simply asks patients if they enjoy items that are presumed to be universally indicative of hedonic capacity, for example, “reading a book, magazine, or newspaper” or “the smell of a fresh sea breeze”. This methodology does not account for the fact that the desirability of these activities is not necessarily related to disease status or anhedonic impairment. It thus may be unsurprising that SHAPS scores were not predictive of RBD classification. Although RBD is unlikely to be the perfect index of hedonic impairment, its ability to objectively assess patients is a strength that measures such as the SHAPS do not possess. 

Given the nascence of the RBD measure, it is difficult to assess its construct validity at this time. However, some degree of construct validity may be implied by the work of Lawlor et al. which was performed on the same EMBARC PRT data [20]. This work showed that the impaired PRT performance of depressed individuals was likely due to an altered evidence accumulation process. Although examining the relationship of these prior findings with RBD was not a goal of this manuscript, doing so may be worthwhile as one would expect high RBD to negatively affect the quality of an individual’s evidence accumulation process (or vice versa).

RBD was not reflected in common sociodemographic features or subjective clinical severity scales, further highlighting the importance of adopting objective symptom measures that have incremental validity into psychiatric care. Interestingly, response rates of sertraline and placebo were similar among reward task engaged participants. However, among reward task disengaged participants, sertraline was more effective than a placebo even after controlling for baseline depression severity, thereby identifying a subgroup of patients with MDD who would uniquely benefit from antidepressant treatment. This has significant clinical implications. High rates of placebo response have resulted in the failure of multiple phase II and III medication trials [21]. RBD may enrich future clinical trials by identifying patients who are less likely to respond to a placebo. Additionally, RBD can be utilized in clinical practice to identify instances of MDD likely to respond equally well to treatments with lower side effects as compared to antidepressant medications, regardless of depression severity. 

### Limitations and Future Directions

This work only assessed the predictive power of RBD regarding response to treatment in an unmedicated, moderate-to-severely depressed sample of MDD patients who were able to tolerate rigorous testing sessions across various days; thus, RBD findings may not extend to other MDD samples. Although promising, validation within this cohort and in future studies in separate cohorts is needed. An interesting direction for future research would be the assessment of RBD as a predictor of differential treatment outcome across several additional treatment paradigms (e.g., different antidepressant medications or medication versus psychotherapy). Additionally, we did not examine the test–retest reliability of RBD for behavioral or outcomes data, which should be a key goal of any future RBD study. This additional validation testing would be reassuring given that RBD did not trend with the SHAPS or QIDS–SR.

## 5. Conclusions

Reward Behavior Disengagement is a novel, objective measure that identifies a new, prognostically significant category of reward impairment in depression. RBD-based classifiers were independent of common sociodemographic and clinical measures, yet uniquely identified differences in acute-phase treatment response to sertraline versus placebo. RBD may represent one of the first clinically relevant neuroeconomic biomarkers of anhedonic impairment.

## Figures and Tables

**Table 1:** Study population demographics.

Features	Healthy Controls	MDD Sample
No. of Participants	40	196 *
Age, mean years (SD)	37.6 (14.9)	37.2 (13.1)
Female (%)	25 (62.5)	129 (66.2)
Race/Ethnicity		
Caucasian (%)	26 (65)	124 (63.6)
African American (%)	9 (22.5)	45 (23.1)
Asian (%)	3 (7.5)	14 (7.18)
Native American/Alaskan (%)	0 (0)	1 (0.51)
Hawaiian/Pacific Islander (%)	0 (0)	0 (0)
Other (%)	2 (5.0)	11 (5.64)
Year of Education, Mean (SD)	15.2 (2.3)	14.9 (2.4)
Number of MDD Episodes (SD)	0 (0)	11.1 ** (20.4)
Age of Onset (SD)	~	16.1 (6.01)

Note: Major depressive disorder (MDD); standard deviation (SD); * the probabilistic reward task data from one of these participants was suggestive of a lab error and thus was omitted from model development; ** eight participants had too many MDD episodes to count and were not included in this entry.

**Table 2:** Demographic associations with reward behavior disengagement status in depression.

Category	Reward Task Disengaged *n* (%)	Reward Task Engaged *n* (%)
Sex (${Χ}^{2}$ = 0.62, *p* = 0.43)		
Male	22 (37.9)	44 (32.1)
Female	36 (62.1)	93 (67.9)
Race (${Χ}^{2}$ = 3.02, *p* = 0.22)		
Caucasian	39 (67.2)	85 (62.0)
African American	15 (25.9)	30 (21.9)
Other	4 (6.9)	22 (16.1)
Employment Status (${Χ}^{2}$ = 3.65, *p* = 0.16)		
Full-time	11 (19.0)	42 (31.3)
Part-time	14 (24.1)	33 (24.6)
Unemployed	33 (56.9)	59 (44.0)
Length of Current MDE (${Χ}^{2}$ = 0.60, *p* = 0.74)		
0–6 months	19 (32.8)	49 (35.8)
7–24 months	14 (24.1)	37 (27.0)
>24 months	25 (43.1)	51 (37.2)
Number of Lifetime MDEs (${Χ}^{2}$ = 0.24, *p* = 0.89)		
<3	15 (27.8)	28 (25.2)
3–5	11 (20.4)	21 (18.9)
>5	28 (51.9)	62 (55.9)
Monthly Income in USD (${Χ}^{2}$ = 4.77, *p* = 0.09)		
<2000	29 (63)	51 (44.7)
2000–4000	11 (23.9)	35 (30.7)
>4000	6 (13.0)	28 (24.6)
Marriage Status (${Χ}^{2}$ = 0.61, *p* = 0.43)		
Married or partnered	10 (17.2)	30 (22.2)
Single, divorced, separated, or widowed	48 (82.8)	105 (77.8)
Education status (${Χ}^{2}$ = 1.80, *p* = 0.62)		
Did not graduate high school	2 (3.4)	3 (2.1)
High school graduate or equivalent	13 (22.4)	37 (25.9)
Some college	17 (29.3)	52 (36.4)
College or advanced degree	26 (44.8)	51 (35.7)
Medical Comorbidities (${Χ}^{2}$ = 1.88, *p* = 0.60)		
None	20 (37.7)	61 (47.7)
1	8 (15.1)	13 (10.2)
2	9 (17.0)	18 (14.1)
3 or more	16 (30.2)	36 (28.1)

Note: Major depressive episode (MDE); United States dollars (USD).

## Data Availability

These data can be accessed on the National Institute of Mental Health Data Archive website (https://nda.nih.gov/edit_collection.html?id=2199, accessed on 30 April 2015).

## Supplementary Materials

The following supporting information can be downloaded at: https://www.mdpi.com/article/10.3390/bs13080619/s1. The Supplementary Materials include a detailed description of how to calculate the Reward Behavior Disengagement (RBD) and associated references [22,23,24,25,26]. Also included are four Supplementary Figures. Supplementary Figure S1: Probabilistic reward task (PRT) paradigm illustrating the task used in this study; Supplementary Figure S2: Comparison of Reward Behavior Disengagement model to signal detection and economic paradigms; Supplementary Figure S3: Task disengagement as modeled by signal detection theory (SDT), economic, and combined (i.e., RBD) paradigms; Supplementary Figure S4: Mean reward learning (ΔRB) and ΔDisc in RBD-based subgroups.
