# Supplementary material for: Reward Behavior Disengagement, a Neuroeconomic Model-Based Objective Measure of Reward Pathology in Depression: Findings from the EMBARC Trial
Source: Behav Sci (Basel). 2023 Jul 25;13(8):619. doi: 10.3390/bs13080619 (PMC10451479; doi:10.3390/bs13080619)
Supplement: Supplementary file 1 [file behavsci-13-00619-s001.zip › behavsci-2455422-supplementary.pdf]

Supplementary Materials

Historical context

Probabilistic reward task (PRT) experiments are rooted in signal detection theory (SDT) paradigms. In traditional SDT experiments, when comparing the utilities associated with different stimuli (in our case "rich" vs. "lean"), the expected value function can be used[22]. With the exception of probability functions, details shown in brackets, [], are those specific to our PRT (Supplementary Figure S1).

$$EV(x_i)=\alpha hp[CD]+\alpha mp[MD]+(1-\alpha)ap[FA]+(1-\alpha)jp[CR] \quad (1)$$

where:
$EV(x_i)$, expected value of a decision criterion [e.g., choosing to identify a mouth as long or short] at signal value x_i [e.g., the image of the mouth];
α, alpha, the base rate or relative probability of encountering a signal from the target distribution [i.e., the number of rewarded rich trials divided by the sum of the number of rewarded rich and rewarded lean trials]; $1 - \alpha$ equals the relative probability of encountering a signal from the foil distribution;
h, benefit of correct detection [5 cents*];
m, cost of missed detection [0 cents];
a, cost of false alarm [0 cents];
j, benefit of correct rejection [5 cents*];
p[CD], probability of correct detection [i.e., correctly identifying rich stimulus];
p[MD], probability of missed detection, equal to 1 − p[CD];
p[FA], probability of false alarm [i.e., incorrectly rejecting lean stimulus];
p[CR], probability of correct rejection, equal to 1 − p[FA]
[per participant probabilities were calculated for each block].[1]

The SDT expected value function is composed of two basic properties: probability (p[CD], p[MD], etc.) and payoff (in the form of either cost or benefit). Importantly, this function assumes that the utility of any given signal is directly proportional to payoff value, in dollars, times the probability of correctly

---

* Participants were given 20 cents for correctly identifying PRT stimuli. However, 5 cent values for *h* and *j* were used when computing RBD in our study, as the RBD computation was derived from the original PRT literature by Pizzagalli et al (2005), wherein a 5 cent reward was used. It is important to note that the value used here is ultimately arbitrary when *a* and *m* are zero and *j* = *h*.

accepting/rejecting the target or foil stimulus. Although this "probability times payoff equals utility" estimation is intuitive, it ignores several landmark advancements in behavioral economics and has been shown to be experimentally invalid[23-25]. However, the SDT expected value is still useful. Unlike traditional economic utility, the SDT expected value function accounts for signal uncertainty when modeling value/utility. An SDT paradigm thus provides insight into how one might implement a similarly useful, but more descriptive model using a modern behavioral economics framework[22].

Derivation of Reward Behavior Disengagement (RBD)

Our study uses a variant of the SDT expected value function. However, instead of using the raw "value" and "probability" values found in our PRT data, these values are first processed using the value-function and the probability weighting-function from Cumulative Prospect Theory[22,26]. These new economically principled values are then used in the standard "expected value" function from SDT (Equation 1)[22]. More information regarding this integrated signal and economic (ISE) value function can be found in the works cited by Lynn et al[22].

For this analysis, the output of the ISE value function represents the amount of money one would theoretically need to pay a participant to choose to identify a stimulus as either rich or lean. As such, one would expect ISE_{Rich} to be less than ISE_{Lean}, given that one would need to pay a participant less to choose the rich stimulus (because it is more frequently rewarded). The ISE_{Rich} and ISE_{Lean} are combined per block and per participant to derive what we call Reward Behavior Disengagement (RBD), which is a performance-based behavioral measure of the tendency of a participant to assign different values to two imperfectly discernible and unequally rewarded stimuli. RBD ranges between 0 and 15. An individual with an RBD of 15 implies that the rich and lean stimuli were indistinguishable from each other in terms of their respective values. An individual with an RBD approaching 0 implies that the rich and lean stimuli were infinitely different from each other in terms of their respective values.

The formula for a participant's per block RBD is as follows:

$$\text{Reward Behavior Disengagement (RBD)} = \frac{1}{\text{ISELean} - \text{ISERich} + \frac{1}{15}} \qquad (2)$$

Conceptualization of Reward Behavior Disengagement (RBD)

A comparison of RBD to more commonly used frameworks is illustrated in Supplementary Figure S2. RBD is built upon an economic explanation of hedonic behavior, namely, that the decision to engage in an activity is contingent upon a favorable cost/benefit analysis, wherein the costs include, at minimum, the effort required to perform the activity well. Reward impairment, as examined from this perspective, can thus arise anytime the effort associated with a normally rewarding activity is overly penalized, and ultimately results in task disengagement (as evidenced by higher than normal RBD). The core behavioral assertions of the RBD model are as follows: 1) task disengagement should increase as the perceived task difficulty increases, and 2) task disengagement should decrease as the subjective value of rewards/losses increases (see Supplementary Figure S3 for illustration).

As previously mentioned, task engagement, as indexed by RBD, is thought to directly impact any measure of reward learning. However, the SDT paradigms, which have previously been used to assess PRT reward learning, do not model task engagement in an economic framework. Specifically, these SDT paradigms can account for signal uncertainty (i.e., the inability of participants to perfectly identify PRT stimuli), but they do not account for the effects of payoff uncertainty (i.e., the inability of participants to perfectly determine which PRT stimulus is more rewarding). Consequently, if modeled by SDT paradigms, RBD predicts that a person's engagement in the PRT is constant, regardless of the magnitude of reward or loss at stake; yet, engagement in a task by any unimpaired agent should increase as the stakes rise. As shown in Supplementary Figure S3, a combination of signal and economic frameworks (i.e., the "Integrated Signal and Economic approach"[22]) enables our RBD metric to account for both signal uncertainty and payoff uncertainty, and thus can better model task engagement.

Other Formulas

$$\text{Response Bias: } \log \mathrm{b} = \frac{1}{2}\log\left(\frac{\text{RichCorrect}\times\text{LeanIncorrect}}{\text{RichIncorrect}\times\text{LeanCorrect}}\right) \tag{3}$$

$$\text{Discriminability: } \log \mathrm{d} = \frac{1}{2}\log\left(\frac{\text{RichCorrect}\times\text{LeanCorrect}}{\text{RichIncorrect}\times\text{LeanIncorrect}}\right) \tag{4}$$

Additional Findings

In order to show potential differences in PRT measures by RBD-based index of engagement (i.e., intact reward learning and discriminability for reward engaged MDD participants), the mean response bias, discriminability, and RBD of the HC and MDD groups were calculated for each block (see above for formulas). Reward learning (ΔRB) was measured by the change in response bias between block 1 and 2. The change in discriminability between blocks (ΔDiscr) was also examined. For ΔRB, a positive difference score would likely indicate an increase in bias in block 2 relative to block 1. For ΔDiscr, a difference score around 0 would indicate no change in discriminability across the blocks. However, please note that traditional PRT measures such as response bias and discriminability share many closely related computational variables with RBD, and comparisons between these measures are difficult to interpret.

Supplementary Figure S4 illustrates the differences in ΔRB and ΔDiscr across groups: ΔRB of HC, reward engaged MDD, and reward disengaged MDD participants differed significantly ($F(2,232) = 7.32$, $p < 0.001$). Specifically, reward disengaged MDD participants ($M = -0.060$, $SE = 0.025$, $N = 58$) had significantly lower ΔRB than both HC ($M = 0.030$, $SE = 0.031$, $N = 40$, $p = 0.026$) and reward engaged MDD participants ($M = 0.056$, $SE = 0.017$, $N = 137$, $p < 0.001$). Of note, ΔRB did not differ between HC and reward engaged MDD participants ($p = 0.449$). Additionally, ΔDiscr differed between HC, reward engaged MDD, and reward disengaged MDD participants ($F(2,232) = 6.10$, $p = 0.003$). Reward disengaged MDD participants ($M = -0.091$, $SE = 0.024$) had significantly lower ΔDiscr than HC ($M = -0.008$, $SE = 0.029$, $p = 0.031$) and

reward engaged MDD participants ($M = 0.011$, $SE = 0.016$, $p = 0.002$), despite a small but significant increase in the ratio of rich stimulus reward probability to lean stimulus reward probability[†].

[†] A one-way ANOVA comparing the effect of group (HC, reward engaged MDD, and reward disengaged MDD) on the mean ratio of rich-to-lean stimulus reward probability showed statistical significance ($F(2,467)$, $p = 0.009$), but accounted for only 2% of the variability.

Supplementary Figures

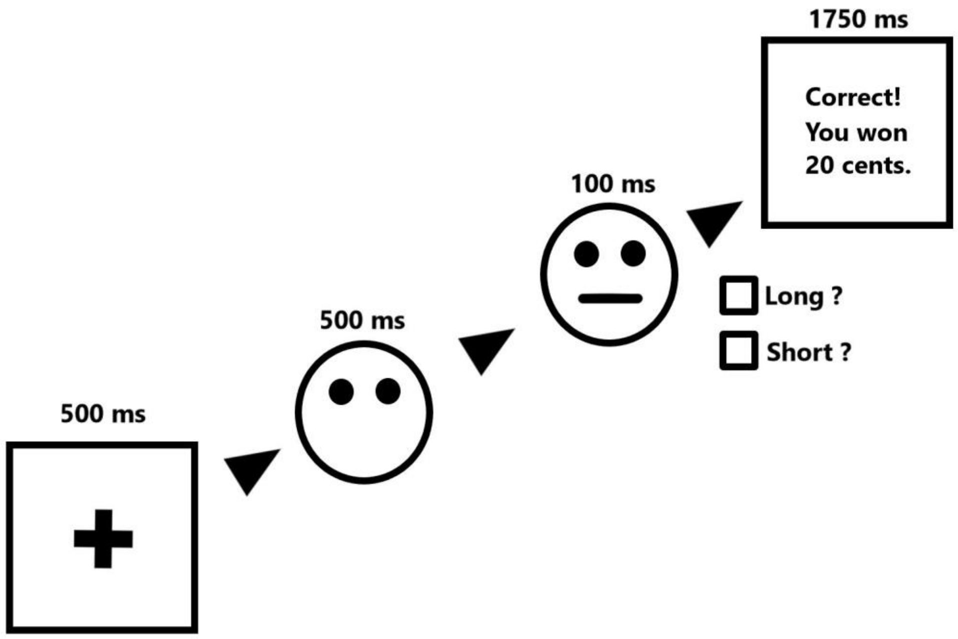

Supplementary Figure S1. Probabilistic reward task (PRT) paradigm illustrating the task used in this study.

Reward Behavior Disengagement combines signal detection and economic paradigms

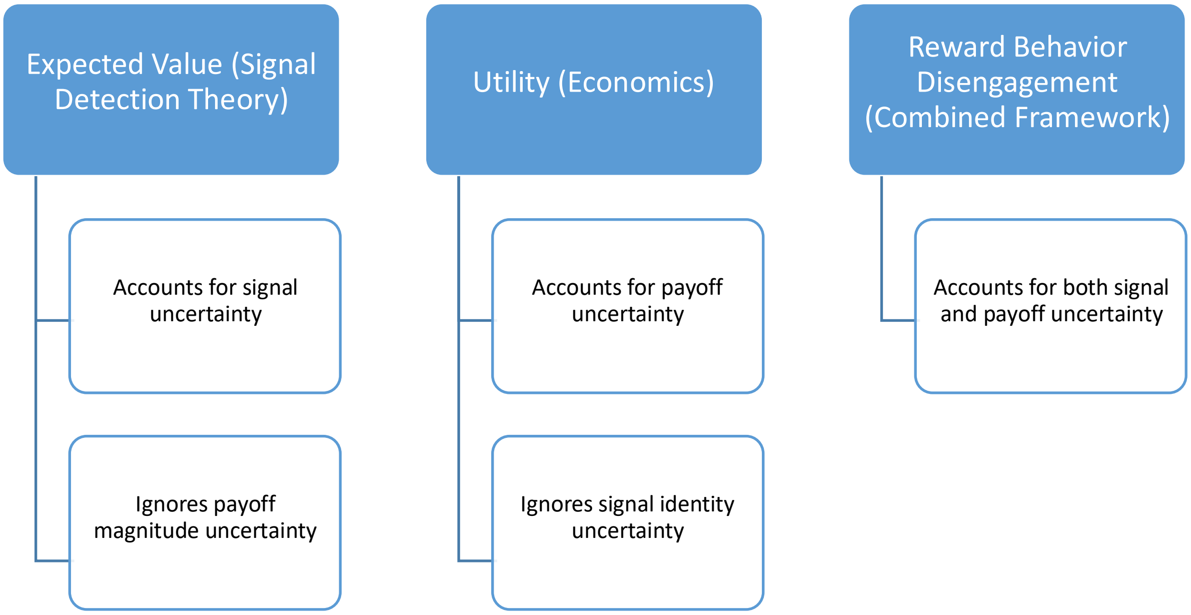

Supplementary Figure S2. Comparison of Reward Behavior Disengagement model to signal detection and economic paradigms. Note: Supplementary Figure S2 depicts how reward behaviors are modeled using decision-making paradigms from signal detection theory (SDT), economics, and combined frameworks. For the probabilistic reward task (PRT), while the SDT expected value function accounts for signal uncertainty (i.e., the difficulty of correctly identifying PRT stimuli), this function does not account for the effects of payoff uncertainty (i.e., the inability of participants to determine which PRT stimuli is more rewarding). The consequences of this are shown in Supplementary Figure S4.

A combined SDT and economic paradigm (i.e., RBD) which models task engagement plausibly

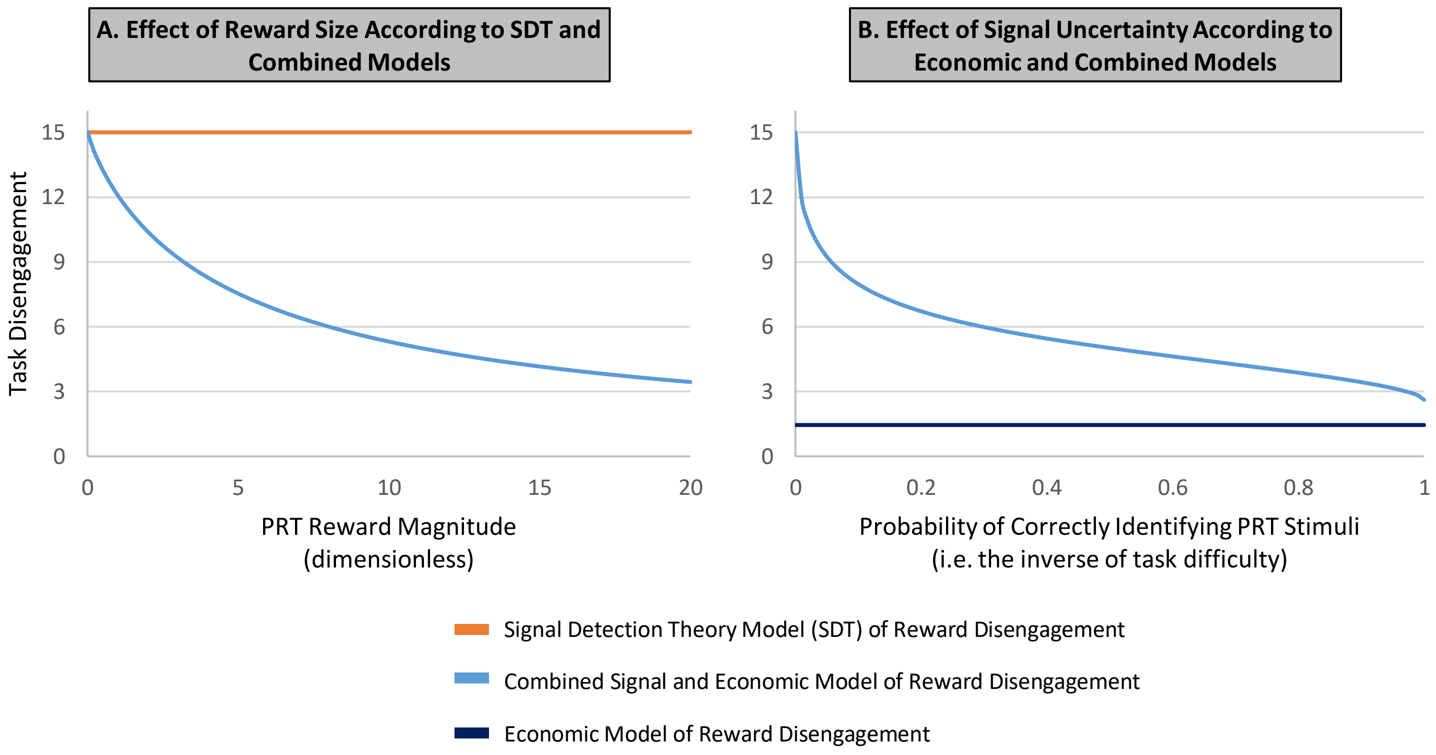

Supplementary Figure S3. Task disengagement as modeled by signal detection theory (SDT), economic, and combined (i.e., RBD) paradigms. Note: Shown here, an SDT-based framework predicts that a person's engagement in the PRT is constant, regardless of the magnitude of the reward at stake; yet, the level of engagement in any task ought to be modified by the magnitude of reward at hand. Similarly, although traditional economic utility accounts for reward magnitude, it does not account for the effect of signal uncertainty on task engagement. A combined approach allows our RBD metric to account for both signal uncertainty and value uncertainty in decision-making behavior. The RBD model asserts that: 1) disengagement should decrease as the perceived value of rewards/losses increases (Panel A), and 2) disengagement should increase as signal uncertainty increases (Panel B). PRT target-to-foil ratio was fixed at 3:2 in these examples.

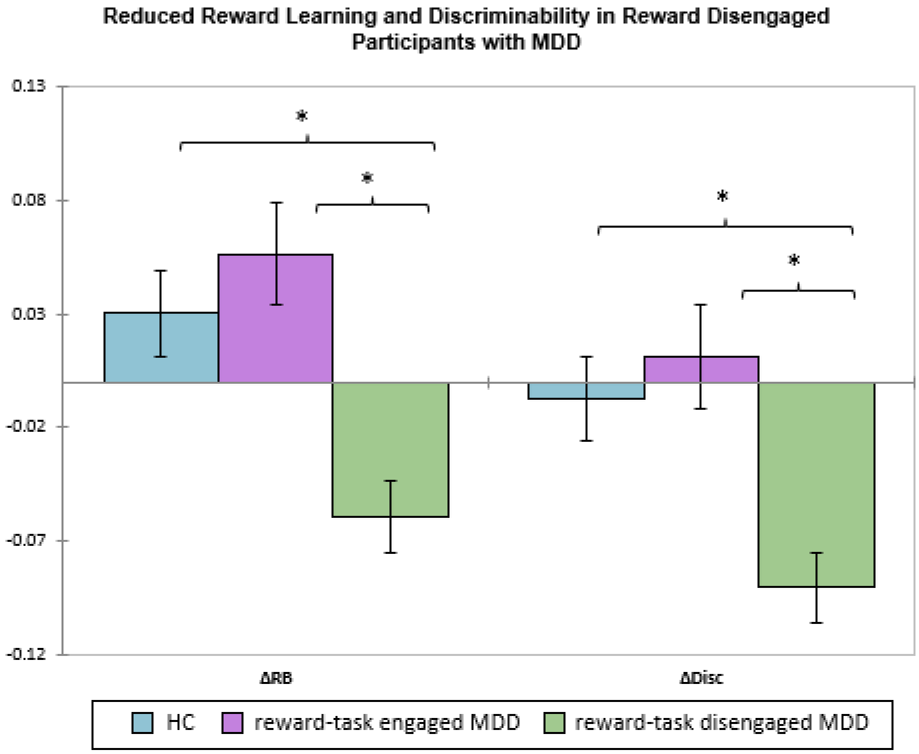

Supplementary Figure S4. Mean reward learning (ΔRB) and ΔDisc in RBD-based subgroups. Note: Shown with standard error bars; major depressive disorder (MDD); healthy control (HC); response bias Δ (ΔRB); discriminability Δ (ΔDisc) between blocks 2 and 1 of the probabilistic reward task (ΔDisc); * $p < 0.005$.

References

22. Lynn, S.K.; Wormwood, J.B.; Barrett, L.F.; Quigley, K.S. Decision making from economic and signal detection perspectives: Development of an integrated framework. *Front. Psychol.* **2015**, *6*, 952. https://doi.org/10.3389/fpsyg.2015.00952.
23. Machina, M.J. Choice under Uncertainty—Problems Solved and Unsolved—Responses. *J. Econ. Perspect.* **1988**, *2*, 181–183.
24. Hansson, H.; Lagerkvist, C.J. Decision making for animal health and welfare: Integrating risk-benefit analysis with prospect theory. *Risk Anal.* **2014**, *34*, 1149–1159. https://doi.org/10.1111/risa.12154.
25. Oliver, A. A quantitative and qualitative test of the Allais paradox using health outcomes. *J. Econ. Psychol.* **2003**, *24*, 35–48. https://doi.org/10.1016/S0167-4870(02)00153-8
26. Tversky, A.; Kahneman, D. Advances in Prospect-Theory—Cumulative Representation of Uncertainty. *J. Risk Uncertain.* **1992**, *5*, 297–323. https://doi.org/10.1007/Bf00122574.
